# Nerve-Storms

**Published:** 1876-10

**Authors:** P. H. Hayes


### NERVE-STORMS.

P. H. HAYES, M. D.

The theory of nerve-storms is that of accumulation and discharge of nerve-force. In the long heated terms of our summer season, the storm forces of nature may long remain silent, but they are not dead. After long waiting, and when the sky above has almost begun to seem like brass, and the earth like iron, there comes a day when the heavy atmosphere seems too oppressive to be borne, and the strain on our nerves calls more and more strongly for relief, and then casting our eyes up to the sky, we see the heavens darken. The dead atmosphere begins to move in wide currents, the clouds grow denser, the lightnings play in the firmament, the rain begins to fall, and after a few hours, the storm has passed; all nature is refreshed, and we breathe a new life. Earth, sky, atmosphere and man are again on good terms. That something so analagous to all this should exist in a certain class of nerve diseases as to have suggested the name of nerve-storms, will appear less wonderful as we proceed in our analysis.

As a type of this class of diseases, epilepsy is one of the best illustrations. Here there is a long period of rest or repose between the attacks, in which every operation of the living organism may be performed as, or nearly, as in perfect health, when all at once there is a sudden and terrifying convulsion, a distortion of features, a prolonged oppression of brain with difficult respiration and unconsciousness, and this state of things slowly falls out into the usual state of health, which may be apparently very good indeed. Now what we have to remark here, is that epileptic fits are not epilepsy—they are the outbreak or expression of the disease. The disease is in the nervous system of the sufferer, impressed it may be by ancestors, who have had the same disease. We can do nothing by treating epileptic fits merely; we must treat the epileptic diathesis or habit of body.

Hysteria is another of the affections which illustrate admirably this type of nerve disorders. A case came under my care, where the patient, an unmarried lady, had been confined to her bed for years. Every few days this patient, from the calmness and repose of perfect apparent health, would start into the most violent convulsions, beating her breast with both hands in rapid alternation as a drummer beats a drum. In a moment, more or less, this would cease, and she would drum upon the bed with the feet in the same violent manner, and this would in like manner cease and be followed by rolling the head from side to side, so rapidly, that the movements could not possibly be counted. All these movements would cease for an instant, and she would begin to throw herself forward and back in the same rapid manner, catching hold of her feet each time or hold of the footboard. I satisfied myself that these attacks were hysterical in character, while they were chorric in expression. A cold plunge bath, holding the patient under—head and all—for a moment, would perfectly cure these paroxysms and bring about the most perfect calm of body and mind.

Asthma fairly belongs in the group of neuroses which I am describing. In nearly all its forms, there is the interval of variable and perfect rest, and the recurring paroxysms, often sudden and so violent in character, that a patient shall be seized with it in sound sleep at two or three o'clock in the morning, and feel that he shall die in a few minutes if not relieved. He calls loudly for fresh air, braces with his arms against some solid object, so as to do all in his power to force the air into his lungs. The face turns livid and takes on an expression of extreme anxiety and distress. In an hour we may find our patient sleeping quietly, so perfectly may the paroxysm disappear.

Angina pectoris is another of the nerve-storm group of diseases. The patient is seized in an instant, and sometimes while engaged in ordinary conversation, with an awful pain in the region of the heart, piercing through the chest to the throat, and to both arms, and he is filled with indescribable anxiety and terror—a few moments of this, and the attack is over, and the patient is himself again. This disease is very common in England—exceedingly rare in this country.

Another disease, very common in France, England and America, known in England as Megrim, in France as Migraine, and in this country more commonly called *sick headache* and *nervous headache*, falls very naturally into the catalogue of nerve-storms. Sometimes this neurosis attacks only one half of the head, and then it is more precisely known as Hemicrania. Some persons are subject to megrim every month, especially women, others have it still oftener, and with great regularity. I had a patient, a professional man, who was sure to have an attack every Sunday, perchance it would postpone a day or half day. A pain will begin in the right temple, and often without overstepping the median line, extend itself and reach its greatest intensity in about five hours. It is greatly aggravated by light, sound, the jar of the floor in walking, and also by every thing which in any way increases the blood-pressure in the brain, such as lifting, stooping, coughing, nay, the very beat of the heart seems to hurt the brain. Among other symptoms, the patients often complain of numbness, giddiness, partial blindness, almost always limited to one eye, and hence some have called it the blind headache.

Time would fail me to give even a synopsis here of more of this group of diseases. I will barely name a few others, among which are spasmodic croup, teething convulsions, epileptic vertigo, the *petit mal* of the French, also tic doloreux, St. Vitus dance, spasmodic dysphagia and somnambulism.

The theory of nerve-storms, as I said in the beginning, is based upon the idea that a morbific element, force or agency somehow accumulates in the system up to a certain amount of tension, and then the paroxysm, whatever its nature, comes on with more or less explosive violence. We can hardly resist the idea of quantity, a something which can accumulate and be exhausted. There are every day analogies to support this notion. How often have we all restrained an almost irresistible laugh when in company, and got so "full" that we could not "hold in" any longer, and had to go away and have an explosion. Grief holds the same analogy. It is well known that this may be restrained for a long time, and finally the sufferer may "break down" in a flood of tears, sobbing and sighing; the grief-storm is over and the sky clears again. How often we are told by our epileptic, asthmatic and magrim patients, that if they have an unusually long interval of rest between their paroxysms, they expect an unusually severe paroxysm. How many times have I heard megrim patients say, "It's no use, your medicines, they only *abate* to *prolong* my suffering; Iv'e *got* to suffer *just so much.*" The true idea of treatment is not to mistake the paroxysm, the storm, for the disease, but treat the tendency to accumulation of the morbific element. Bearing upon the idea of accumulation and discharge, Dr. Salter observes that each attack of asthma seems to impart for a time, immunity from a repetition of the fit. A patient may expose himself to the exciting causes of the disease immediately after a fit with impunity, and yet as the time for a fit has nearly expired, the slightest imprudence will bring it on.

All of this group of diseases have certain common characters; thus they are all inheritable, and more commonly than otherwise, they are in fact inherited. To show again the wonderful alliance in all these neuropathic disorders, it has been found that through inheritance they undergo transformations into each other. Dr. Salter mentions that a patient of his, and a medical man, made this statement: "My great grandfather and a great aunt had *asthma.* One of my brothers has it. My father had *neuralgia* for many years, ending in *paralysis.* One of my brothers had *spasmodic dysphagia,* another had *epileptic fits,* and several of our family are *somnambulists.*" Moreau mentions a woman who suffered from megrim, her son from epilepsy. In another case megrim in the father was represented in one son by magrim, and in two daughters by neuralgia attacks. Another woman had megrim, and one of her brothers had paroxysms of *insanity,* and another had *asthma.* Strangely enough, these diseases undergo transformation in the same individual. Thus Mrs. H. had megrim occurring at the catamenial period for twenty-three years, and at the end of this period she was seized with well-marked epilepsy at exactly the same periods. In another case gastralgia and megrim underwent *alternations* with some regularity; one month, there would come the old megrim, the next an attack of gastralgia. In harmony with all this, it has been found that the *suppression* of an habitual megrim is liable to be followed by some of the other nerve storms, such as angina pectoris or tic doloreux.

In my next article, I propose to take up the treatment of the neuropathic disorders, which I have here described under their new title of "Nerve-Storms."

